# Examining the Relationship between Gender Contentedness and Sex-Related Experiences among Taiwanese Youth

**DOI:** 10.3390/ijerph182010635

**Published:** 2021-10-11

**Authors:** Chia-Yi Liu, Jen-Hao Kuo, Ting-Hsuan Lee, Carol Strong, Meng-Che Tsai, Chih-Ting Lee

**Affiliations:** 1 School of Medicine, College of Medicine, National Cheng Kung University, Tainan 70101, Taiwan; i54066518@mail.ncku.edu.tw (C.-Y.L.); thomas42913@gmail.com (J.-H.K.); asdsky33lee@gmail.com (T.-H.L.); 2 Department of Public Health, National Cheng Kung University Hospital, College of Medicine, National Cheng Kung University, Tainan 70101, Taiwan; carol.chiajung@gmail.com; 3 Department of Pediatrics, National Cheng Kung University Hospital, College of Medicine, National Cheng Kung University, Tainan 70101, Taiwan; 4 Department of Family Medicine, National Cheng Kung University Hospital, College of Medicine, National Cheng Kung University, Tainan 70101, Taiwan

**Keywords:** gender contentedness, sexual health, risky sex behavior, sexually explicit media, adolescent

## Abstract

Little is known about how gender contentedness is related to sex-related experiences among Taiwanese adolescents. Secondary analysis of data (*n* = 2624, Mage = 13.3 ± 0.47 years and 51% males) on a longitudinal youth cohort was used to track the evolving development of sex-related experiences such as exposure to sexually explicit media, romantic experiences, and sexual behaviors. Hierarchical multinomial logistic regression analyses were applied to study the effects of gender contentedness on sex-related outcome variables. The results showed that nearly 10% of the subjects were not contented with their gender. As compared to peers, gender-discontented adolescents had a higher likelihood of exposures to sexually explicit media (odds ratio [OR]. = 1.70, 95% confidence interval [CI]. = 1.18–2.46) and risky sex behaviors (OR = 2.22, 95% CI = 1.03–4.81). These results shed light on the impact of self-perceived gender contentedness on sex-related experiences in Taiwanese adolescents. Our findings are helpful for the development of practical guidance on sexual health issues, particularly for those who are discontented with their gender.

## 1. Introduction

Gender identity develops in early childhood and becomes more flexible and constantly shaped during adolescence, as individuals simultaneously undergo a series of physical, psychological, contextual and sexual changes, where the factors of family, peers, individuals and society play a critical part [1,2]. During this time, adolescents start to explore romantic relationships and seek sexual experiences, although these behaviors may not be socially desirable at this stage. They usually get access to sex-related information through such diverse approaches as learning from their parents, their peers, school health education, or alternative sources including the Internet (e.g., sexually explicit websites) [3]. Some argue that viewing sexually explicit materials is related to the formation of gender norms and social roles, particularly for gender minority groups [4]. However, pubertal transition, deficient parental control, and insufficient social support along with non-conforming gender identity, might intensify the instability of adolescence and contribute to risky sexual behaviors [5,6,7,8]. Risky sexual behaviors including early sexual debut and unsafe sex are reported to be associated with long-term negative impacts, especially health issues such as the acquisition of sexually transmitted infections, unintended pregnancy, and substance abuse (e.g., drugs, alcohol, and tobacco) that are prevailing health problems among adolescents who conduct risky sexual behaviors [9,10].

The construction of identity and expression of gender is thought to be a life-long process that typically involves the formation of gender norms via seeing, learning, and behaving in expected ways [11]. During this process, gender identity and expression can be fluid and comprise a wide range of characteristics and behaviors that are not necessarily described as masculine or feminine [12]. Generally speaking, gender non-conforming adolescents may undergo more psychological struggles in confirming their gender identity as compared to their gender conforming peers [13]. Past research found that gender nonconformity may be linked to poor psychological health, low self-esteem, and even less peer acceptance [14,15]. As adolescence is a crucial time for socialization and access to sexual issues, accumulating evidence from research conducted in Western societies has demonstrated that gender minority adolescents may be at risk for exposure to sexually explicit materials, passive engagement in sexting experiences, and risky sexual behaviors [16,17,18]. Because of limited data from outside of the United States and Western context, it remains unclear about the ways in which gender identity/conformity is linked to sex-related experiences among Taiwanese adolescents.

Given that gender identity is a sensitive issue in adolescence, it is not easily assessable in an epidemiological survey. Egan et al [19] argued that gender-atypical behavior and dissatisfaction with one’s gender assignment may co-occur in gender nonconforming individuals, suggesting self-perceived typicality and contentedness to be two essential dimensions and reliable indices of a common and more global underlying factor of self-perceived gender compatibility. Prior research has revealed that self-perceived gender compatibility may be associated with concurrent and longitudinal psychosocial adjustment among children and adolescents [20,21,22]. Self-perceived typicality evolves with cognitive maturation that enables children to compare themselves to their same-sex peers in the ways they behave, while self-perceived contentedness forms after gender membership develops and it is subject to satisfaction with perceived sex-typed attributes [23,24,25]. That said, gender contentedness itself can be seen as an early form of gender identity and serve as a useful surrogate variable measuring gender conformity [25], when the perception of gender identity/conformity is not yet fully concrete in children and adolescents.

Therefore, operationalizing gender conformity as perceived gender contentedness, this study aimed to explore the relationship between gender contentedness and sex-related experiences such as media exposure, romantic experience, and sex behaviors in a longitudinal cohort of Taiwanese youth.

## 2. Materials and Methods

Publicly accessible data were retrieved from the Taiwan Youth Project (TYP) (http://www.typ.sinica.edu.tw (accessed on 1 July 2020)), which was launched by the Institute of Sociology, Academia Sinica, Taiwan. In brief, this project used a school-based, stratified sampling design to survey 7th and 9th graders in 40 schools in Taipei City (16 schools), Taipei County (15 schools), and Yilan County (9 schools). [26]. In the study, only 2624 adolescents (Mage = 13.3 ± 0.47 years) in the 7th grade (wave 1) who replied partially to the questions about gender contentedness, pubertal timing, and sexually relevant questions were deemed valid for the analysis. Subjects were followed annually until 2009 (wave 9), although some waves were not exactly one year apart [27].

### 2.1. Measures

#### 2.1.1. Gender Contentedness (Wave 1)

Gender contentedness was defined by a self-reported question “Are you satisfied with your gender?” The answers were rated on a 4-point Likert scale from 1 (very dissatisfied) to 4 (very satisfied). For analytical purposes we further dichotomized the answers into two groups: gender-discontented (GD, scores 1 and 2) and gender-contented (GC, scores 3 and 4).

#### 2.1.2. Sexual Media Exposure (Wave 2)

Exposures to adult-only or restricted media were assessed using one question: “Have you ever seen any of the following adult-only or restricted media?” We calculated counts of the chosen modalities, including websites, magazines, comic books, novels, films, and other (with a range from 0 to 6). The use of this composite scale has been justified in a Taiwanese social setting, given the Chinese wording of the question referring to sexually explicit content (e.g., sexual intercourse and nudity) [27]. For the analytical purposes we further subdivided the participants based on the counts of modalities that they were exposed to into three groups: “never”, “1–2 kinds”, and “3–6 kinds”.

#### 2.1.3. First Romantic Experience (Waves 2–9)

Based on the serial self-reported status of romantic relationships (i.e., Have you been in a romantic relationship?) from waves 2 to 9, the first romantic experience was identified and then categorized into four starting time points: “junior high school”, “senior high school”, “college”, and “not as of yet being in a relationship” [28].

#### 2.1.4. Age at Sex Debut (Wave 9)

The age at sex debut was self-reported at wave 9. According to their reports, we split the participants into “early” and “late” two groups using a cutoff of age at 16 and 18 years. The chosen cutoff age of early sexual debut was aligned with the legal age for consensual sex and marriage respectively in Taiwan [29].

#### 2.1.5. Unsafe Sex (Wave 9)

Whether having sexual behaviors with condoms or not was used as an indicator of sexual safety. Self-reports on the question “Do you use condoms when you have sex?” were used to categorize the participants into two groups: “having sex with a condom” and “having sex without a condom” [27].

### 2.2. Covariates (Wave 1)

Pubertal timing was measured according to the Chinese version of the Pubertal Developmental Scale via a subjective evaluation of physical changes, including growth spurts, body hair development, skin changes, breast growth, voice change, and menarche/facial hair growth, on a 4-point Likert scale. Unlike the rest of items, menarche was evaluated dichotomously (“yes” or “no”) and recoded into point 1 and point 4 respectively. We summarized the PDS scores with a higher score representing an earlier timing of sexual maturation. Aligned with previous research, the PDS scores were standardized within the same sex and gender groups [30]. Further, we classified the participants into three groups: early-puberty (more than 1 standard deviation above), on-time puberty (within 1 SD either way), and late-puberty (more than 1 SD below).

Monthly family income and administration districts were used to reflect the socioeconomic status of the subjects. Family income was subdivided into three levels: “New Taiwan Dollar (NTD) 30,000 or less”, “NTD 30,001–60,000”, and “NTD 60,001 or more”, and residence was categorized into three districts: “Taipei City”, “Taipei County”, and “Yilan County”.

In addition, self-reported parental relationships were used as a proxy for family supports, while social networks were indicated by their friend counts. We combined two four-point questions on parental relationships, one for the relationship with their father and the other for that with their mother, to form a single score ranging from 2 to 8 and then reclassified subjects into two groups: “satisfied” (from 6 to 8) and “dissatisfied” (from 2 to 5). The number of friends was subdivided into three levels: “none”, “1 to 3 friends”, and “4 or more friends”.

### 2.3. Statistical Analysis

The demographic characteristics of the subjects were summarized using descriptive analysis. The χ^2^ and independent t tests were applied to compare categorical and continuous variables between groups. Furthermore, we examined the relationship between gender contentedness and sex-related experiences in a gender-stratified approach using hierarchical multinomial logistic regression analyses. Model 1 tested the univariate association between gender contentedness and outcomes using GC adolescents as the reference group. Model 2 included monthly family incomes and administrative districts as covariates. Model 3 further adjusted for pubertal timing and covariates in Model 2; Model 4 was the fully adjusted model including all potential covariates. Odds ratios (ORs) with 95% confidence intervals (CIs) were reported to determine statistical significance

## 3. Results

Among surveyed adolescents in the 7th grade, with 51.4% (*n* = 1348) being male, of the 2624 students included in the final analysis, 90.9% (*n* = 2385) were satisfied with their gender. As compared to GC adolescents, GD adolescents were more advanced in pubertal development, less satisfied with parental relationships, and had fewer friends (Table 1). Comparing outcome variables between GD and GC participants, GD adolescents reported having access to more modalities of sexually explicit media and a higher proportion of GD adolescents reported having their first romantic relationship before age 18 (Table 2).

In unadjusted regression analyses (Model 1), GD adolescents were inclined to be exposed to 3-6 kinds of sexually explicit media (OR = 1.70, 95% C I= 1.18–2.46) (Table 3). This association remained significant in Model 2, considering family income and administrative district. However, the significance of the association was not present after adjusting for pubertal timing, parental relationship and friend counts. In females, GD adolescents were likely to be exposed to 3-6 kinds of sexually explicit media (OR = 2.68, 95% CI = 1.67–4.30). This relationship remained significant in adjusted models. Contrarily, we did not observe the association between gender contentedness and sexually explicit media exposure in males.

Furthermore, there was no difference in reports of the first romantic relationship before age 18 (Table 4). After entering college, GD adolescents were less likely to engage in their first romantic relationship (OR = 0.48, 95% CI = 0.25–0.93). Stratifying the analysis by gender, we found GD females were less likely to engage in their first romantic (OR = 0.47, 95% CI = 0.23–0.95), but not males.

Additionally, there was no significant difference between GC and GD adolescents in early sex debut, irrespective of whether it was defined by occurrence before 16 or 18 years (Table 5). However, GD adolescents had a higher likelihood of experimenting with sexual intercourse without condoms (OR = 2.22, 95% CI = 1.03–4.81) as compared to their GC peers.

## 4. Discussion

Our study was, to the best of our knowledge, the first to explore the longitudinal relationship between gender contentedness and sex-related experiences among Taiwanese youth. We found that nearly 10% of Taiwanese adolescents were not contented with their gender; this is similar to the rate of gender minority in American adolescents [31]. As compared to their peers contented with gender, GD adolescents were more likely to gain access to sexually explicit media and have unprotected sex. However, this relationship was not significant after covariates were controlled for. The implications of our study are provided as follows.

We found GD adolescents in our sample, particularly girls, were more likely to gain access to sexually explicit media. This can be explained as sexually explicit media serving as learning material for gender norms and sex behaviors. Thereby, longer and more frequent exposure to sexually explicit online media is found more commonly among youth in some sexual and ethnic minorities [5,6]. As social media is thriving in popularity among the new generations, young people receive diverse information, including sex-related content. Social media thus has a great impact on sex-related attitudes and behaviors among youth [32]. Despite the results showing that GD youth had a higher likelihood of being exposed to sexual social media, we did not evaluate the possibility of this potential avenue for relaying sexual information to youth. Considering the diversity of social media that adolescents get access to, it should be perceived by the educational authorities as one of the potential routes for educational intervention for adolescents so that proper sexual knowledge can be provided, thus reducing the anxiety of sexually minor youths [3].

Our results did not reveal any difference in the rates of entering romantic relationships before college between GC and GD adolescents. Under the cultural context in Taiwan, school education and family parenting usually emphasize academic performances and prohibit romantic relationships among adolescents [33]. However, as access to the Internet increases, GD youth tend to initiate romantic relationships online earlier during adolescence as compared to their GC peers, because of the deficiency of friendship support and parental monitoring offline [34]. We also found that GD subjects in adulthood had more difficulty in entering romantic relationships. This might be associated with a conservative cultural orientation in East Asian countries such as Taiwan, especially among the generations of parents. Entering and existing romantic relationships are sometimes influenced by parental factors [35]. Taiwanese parents with a conservative attitude toward diverse gender identities may impose stress and restrain the freedom to select a partner among their sexually minor children [36,37].

In Taiwan, almost all adolescents live with their families and feel a general lack of privacy, which plays a critical role in the relatively lower rate of premarital sex than that in Western societies [38,39]. This may also explain why there was no significant difference in early sexual debut between GC and GD adolescents in our analysis. Although adolescents were influenced in their sexual permissiveness and attitudes, thus increasing the odds of sexual behaviors, it is notable that limitations due to social norms and their parents may prevent adolescents from engaging in early sexual behaviors [7,8,40]. Nevertheless, our results showed that GD adolescents had a greater tendency to have sex without protection. This stands in line with some previous reports that sexual minority adolescents of both genders may demonstrate a higher likelihood of engaging in risky sexual behaviors, including early sexual debut, no condom use, and anal sex [41,42,43]. There is also some evidence showing the predictive role of viewing sexually explicit online media in subsequent unprotected sex in sexual and gender minority men [44,45]. This tendency may be explained by the emotional oppression commonly felt in sexual and gender minority youth and consequent seeking of an emotional outlet for intimacy development [46]. Additionally, media exposure is viewed as a leading contributor to risky sexual behavior, and evidence shows that delaying exposure to sexual media may decrease the odds of intercourses without condoms [27,47]. The influences of the Internet in this context may deserve further exploration in order to make up for this deficiency in our research and enhance the complete comprehension of sex-related experiences in Taiwanese adolescents.

## 5. Limitations

Our sample has its strength in being a longitudinal cohort study design with a large number of participants and a cluster-based sampling strategy. However, interpretation of the results should proceed cautiously in the presence of some methodological limitations. A significant one is the single-item measurement of perceived gender contentedness, in that it is more like a cognitive evaluation of the importance of gender in an individual’s conception of self [48]. This item may not be able to capture the multidimensionality of gender and gender identity, as it may reflect a mixed satisfaction level with assigned sex at birth, gender role, and gender expression. Despite this, a single-item gender contentedness score still represents an overall perceived feeling toward one’s collective gender, and its simplicity has merit, particularly in longitudinal epidemiological surveys where a more complex construct of detailed questions may not be feasible. Moreover, sex-related experiences may be underreported because of social undesirability, particularly when surveyed before adulthood. Thus, our results can only cast a preliminary glimpse at the evolving development of sex-related experiences between GC and GD adolescents in Taiwan. The complicated mechanisms underlying the association may require further research and analyses.

## 6. Conclusions

In conclusion, GD adolescents are exposed to more sexually explicit media and, eventually, more risky sexual behaviors. Further investigation should address how these sex-related experiences impact adolescent health and development, although this is outside of the scope of the present study. Among the surveyed experiences, sexual risk-taking is of particular concern given its salient association with infection by sexually transmitted diseases. From the perspective of preventative medicine and public health, early prevention is presumed to be more efficient than later treatment of disease. Our preliminary results can hopefully raise awareness about the need for developing practical guidance on monitoring and preventing any negative influences associated with gender nonconformity in the sexual practices and health of developing youth. It may be imperative to integrate the stakeholders of adolescent health, such as their parents, teachers, and health care providers, into promotion of gender equality and development. Moreover, given that the findings may be limited because of our use of a single-item measurement of gender contentedness, we also encourage more studies in order to verify our findings across diverse cultural settings.

## Figures and Tables

**Table 1:** Demographic information of participants at baseline.

Variables, *n* (%)	Gender-Contented (*n* = 2385)	Gender-Discontented (*n* = 239)	*p* Value
Age (years), mean (±SD)	13.30 (±0.47)	13.30 (±0.47)	0.88
Gender			<0.001
Male	1297 (54.4)	51 (21.3)	
Female	1088 (45.6)	188 (78.7)	
Pubertal development scale at W1 ^a^	10.43 (±2.35)	11.22 (±2.25)	<0.001
Administration district			0.18
Taipei City	932 (39.1)	100 (41.8)	
Taipei County	920 (38.6)	98 (41.0)	
Yilan County	533 (22.3)	41 (17.2)	
Monthly family income (NTD)^a^			0.14
<30,000	372 (18.0)	33 (16.4)	
30,001–60,000	899 (43.4)	76 (37.8)	
>60,001	800 (38.6)	92 (45.8)	
Parental relationship ^a^			<0.001
Satisfied	1981 (83.9)	150 (63.0)	
Dissatisfied	381 (16.1)	88 (37.0)	
Friend counts ^a^			<0.001
none	31 (1.4)	7 (3.0)	
1–3 friends	301 (13.2)	50 (21.6)	
4 and above more friends	1947 (85.4)	175 (75.4)	

^a^ Missing data were not included in the denominator. SD indicates standard deviation; NTD, New Taiwan Dollar.

**Table 2:** Comparison of sex-related experiences between gender-satisfied and gender-dissatisfied adolescents.

Variables, *n* (%)	Gender-Contented (*n* = 2385)	Gender-Discontented (*n* = 239)	*p* Value
Sexual media exposure at W2 ^a^			0.016
Never	1152 (50.0)	103 (44.0)	
1–2 kinds	842 (36.6)	84 (35.9)	
3–6 kinds	309 (13.4)	47 (20.1)	
First romantic experience ^a^			0.049
Junior high school	492 (32.9)	53 (36.3)	
Senior high school	329 (22.0)	39 (26.7)	
College	251 (16.8)	12 (8.2)	
Never being in a relationship	423 (28.3)	42 (28.8)	
Sex debut before age 16 years ^a^			0.372
Yes	133 (7.4)	16 (9.1)	
No	1660 (92.6)	159 (90.9)	
Sex debut before 18 years ^a^			0.139
Yes	440 (24.5)	34 (19.4)	
No	1353 (75.5)	141 (80.6)	
Unsafe sexual behavior ^a^			0.054
Having sex with condoms	367 (86.2)	28 (73.7)	
Having sex without condoms	59 (13.8)	10 (26.3)	

^a^ Missing data were not included in the denominator.

**Table 3:** Multinominal regression analysis on the association between gender contentedness and sexually explicit media exposures, OR (95%CI).

	1–2 Kinds				3–6 Kinds			
	Model 1	Model 2	Model 3	Model 4	Model 1	Model 2	Model 3	Model 4
Overall	1.12 (0.83–1.51)	1.08 (0.722–1.62)	1.08 (0.72–1.61)	1.06 (0.70–1.59)	1.70 * (1.18–2.46)	1.62 * (1.00–2.61)	1.52 (0.94–2.48)	1.33 (0.81–2.21)
Males	0.76 (0.38–1.54)	0.97 (0.38–2.47)	0.94 (0.37–2.40)	0.87 (0.33–2.24)	1.90 (0.97–3.71)	1.62 (0.63–4.13)	1.48 (0.57–3.83)	1.16 (0.42–3.18)
Females	1.33 (0.94–1.87)	1.22 (0.77–1.93)	1.23 (0.77–1.94)	1.23 (0.77–1.96)	2.68 ** (1.67–4.30)	2.66 ** (1.45–4.85)	2.55 ** (1.38–4.71)	2.22 * (1.18–4.16)

Model 1 tested the univariate association between gender contentedness and outcomes; Model 2 adjusted for monthly family incomes and administrative districts; Model 3 adjusted for pubertal timing and covariates in Model 2; Model 4 adjusted for parental relationship, friend counts and covariates in Model 3. Gender-contented adolescents were treated as the reference group. OR indicates odds ratios; CI, confidence interval. * *p* < 0.05, ** *p* < 0.01.

**Table 4:** Multinominal regression analysis on the association between gender contentedness and the first romantic relationship.

	Junior High School				Senior High School				College			
	Model 1	Model 2	Model 3	Model 4	Model 1	Model 2	Model 3	Model 4	Model 1	Model 2	Model 3	Model 4
Overall	1.09 (0.71–1.66)	0.75 (0.42–1.33)	0.73 (0.41–1.31)	0.74 (0.41–1.34)	1.19 (0.75–1.89)	0.83 (0.45–1.53)	0.83 (0.45–1.53)	0.87 (0.46–1.62)	0.48 * (0.25–0.93)	0.38 * (0.16–0.90)	0.37 * (0.16–0.89)	0.40 * (0.17–0.97)
Males	2.36 (0.92–6.09)	2.07 (0.62–6.84)	2.17 (0.64–7.30)	2.82 (0.77–10.32)	1.67 (0.57–4.91)	1.05 (0.25–4.41)	1.05 (0.25–4.43)	1.83 (0.37–9.00)	0.31 (0.04–2.59)	NA	NA	NA
Females	0.89 (0.54–1.47)	0.52 (0.26–1.06)	0.53 (0.26–1.09)	0.48 (0.23–1.00)	1.14 (0.67–1.93)	0.83 (0.41–1.70)	0.84 (0.41–1.73)	0.83 (0.40–1.73)	0.47 * (0.23–0.95)	0.43 (0.17–1.07)	0.46 (0.18–1.17)	0.48 (0.19–1.23)

Model 1 tested the univariate association between gender contentedness and outcomes; Model 2 adjusted for monthly family incomes and administrative districts; Model 3 adjusted for pubertal timing and covariates in Model 2; Model 4 adjusted for parental relationship, friend counts and covariates in Model 3. Gender-contented adolescents were treated as the reference group. OR indicates odds ratios; CI, confidence interval; NA, not available. * *p* < 0.05

**Table 5:** Multinominal regression analysis on the association between gender contentedness and age at sex debut and unsafe sex, OR (95%CI).

	Age at Sex Debut								Unsafe Sex			
	Before Age 16 Years				Before Age 18 Years				Having Sex without Condoms			
	Model 1	Model 2	Model 3	Model 4	Model 1	Model 2	Model 3	Model 4	Model 1	Model 2	Model 3	Model 4
Overall	1.26 (0.73–2.16)	1.37 (0.69–2.75)	1.37 (0.68–2.75)	1.35 (0.67–2.75)	0.74 (0.50–1.10)	0.74 (0.44–1.23)	0.75 (0.45–1.25)	0.70 (0.411.18)	2.22 * (1.03–4.81)	2.58 (0.99–6.72)	2.88 * (1.09–7.65)	2.10 (0.76–5.85)
Males	2.26 (0.85–6.04)	2.48 (0.68–9.06)	2.56 (0.69–9.46)	3.09 (0.80–12.01)	0.94 (0.43–2.05)	0.84 (0.29–2.43)	0.84 (0.29–2.44)	0.83 (0.28–2.48)	1.94 (0.38–9.96)	3.31 (0.55–19.81)	5.01 (0.78–32.41)	2.66 (0.34–20.97)
Females	1.03 (0.53–2.01)	1.09 (0.47–2.56)	1.02 (0.43–2.41)	0.99 (0.42–2.37)	0.87 (0.54–1.38)	0.83 (0.45–1.52)	0.82 (0.45–1.51)	0.74 (0.40–1.37)	2.51 (0.99–6.33)	2.31 (0.70–7.61)	2.19 (0.65–7.34)	1.93 (0.54–6.88)

Model 1 tested the univariate association between gender contentedness and outcomes; Model 2 adjusted for monthly family incomes and administrative districts; Model 3 adjusted for pubertal timing and covariates in Model 2; Model 4 adjusted for parental relationship, friend counts and covariates in Model 3. Gender-contented adolescents were treated as the reference group. OR indicates odds ratios; CI, confidence interval. * *p* < 0.05.

## Data Availability

The TYP dataset is archived in the Survey Research Data Archive, managed by the Institute of Sociology, Academia Sinica, Taiwan. It requires registration when accessing to the dataset, although it is free and open to the public. All the waves can be found in the following link: https://srda.sinica.edu.tw/browsingbydatatype_result.php?category=surveymethod&type=2&csid=1.
